# Satisfaction With Community Health Education Among Residents in China: Results From a Structural Equation Model

**DOI:** 10.3389/fpubh.2022.905952

**Published:** 2022-07-11

**Authors:** Yuting Tong, Haipeng Wang, Kangming Zhu, Hanhan Zhao, Yangrui Qi, Jiahui Guan, Yuanyuan Ma, Qiyu Li, Xinying Sun, Yibo Wu

**Affiliations:** ^1^Centre for Health Management and Policy Research, School of Public Health, Cheeloo College of Medicine, Shandong University, Jinan, China; ^2^NHC Key Lab of Health Economics and Policy Research, Shandong University, Jinan, China; ^3^School of English, Shandong Agricultural University, Taian, China; ^4^School of Humanities and Health Management, Jinzhou Medical University, Jinzhou, China; ^5^School of Public Health, Peking University, Beijing, China

**Keywords:** health education, satisfaction, structural equation model, community residents, China

## Abstract

**Background:**

Although community health education has drawn lots of attention from the public, evidence on resident satisfaction is still sparse. This study aims to explore the relationships among five dimensions (perceived quality, perceived value, public expectation, public trust, and public satisfaction) of satisfaction with community health education among Chinese residents.

**Methods:**

We constructed a theoretical public satisfaction model for community health education based on the American Customer Satisfaction Index (ACSI) model. There are five dimensions in the theoretical model, including public expectation, perceived quality, perceived value, public satisfaction, and public trust. We recruited 474 respondents from a quota sampling based on gender and age, and collected information on five dimensions of satisfaction with community health education. The relationships of the five dimensions were examined using structural equation model.

**Results:**

The mean scores of public expectation, perceived quality, perceived value, public satisfaction, and public trust for the participants were 11.44 (total 15), 123.89 (total 170), 14.18 (total 20), 10.19 (total 15), and 15.61 (total 20), respectively. We obtained a structural equation model with a good fitting degree. There was a direct effect of perceived quality on perceived value (γ = 0.85, *P* < 0.01), public trust (γ = 0.81, *P* < 0.01) and public satisfaction (γ = 0.58, *P* < 0.01), and a direct effect of public expectation on public satisfaction (γ = 0.36, *P* < 0.01) and perceived value (γ = 0.25, *P* < 0.01).

**Conclusions:**

We provide a good tool to measure public satisfaction with community health education, which can be potentially used to measure public satisfaction and improve the effectiveness of health education.

## Introduction

Health education provides learning opportunities for both individuals and communities to acquire the necessary information or skills for improving health level of the population (1). The purpose of health education is for the public to increase health knowledge, improve health attitude, and cultivate healthy lifestyle behavior (2). Community health education is an effective way to improve health knowledge and literacy. Health education expertise is critical to developing tailored contents and approaches appropriate to promoting pro-health attitudes and behaviors (3). Access to effective health education can change potential health behaviors. On the contrary, inadequate health education can inhibit the ability of making good health decisions. It has been demonstrated that motivational educators and interactive sessions can lead to good engagement of community health education and thereby contribute to good acquisition of health knowledge and literacy (4).

In order to improve public health literacy, some countries have focused on the effective use of community health education. These efforts include policy introduction, infrastructure improvement, and personnel allocation. In Japan, the Ministry of Health and Labor launched a national policy in 2008 to require local governments to address the shortcomings of their health communication and improve health education systems (5). In the USA and UK, the focus of health education was even more down to communication among the patients and the health-care provider (6, 7). The USA have sought tailoring health communication and community interventions to bridge the gap between behavioral science research tradition with health communication and related behavior-change efforts in the Latino community (8). In Saudi Arabia, heightening recognition of the importance of health educators and considerable efforts from governmental and private health organizations led to numerous milestones in the management, administration and leadership of the health education field (9).

Chinese central government launched Basic Public Health Services (BPHS) in 2009, in which health education was one of the most important programs. Health education is freely provided by community health centers for all residents to disseminate health information and knowledge, which includes five items: provision of health education materials, propagandizing columns, health counseling, health lectures, and personalized health education (10–12). These items are mainly conducted as the form of health communication in communities. However, the delivery of community health education is still suboptimal in most regions in China. The current communication materials do not meet the health needs of local residents in content, format and approach, so the residents have no interest in receiving health education activities (13). Several studies reported poor use of and low satisfaction with community health education. In most provinces or regions, around 60% of residents had use of some items of community health education (14–17). A study in the Hubei province reported that only 47.2% residents were satisfied with community health education (13).

Good satisfaction is very important for effective health education. The success of community health education in BPHS depends on the number of people health educators attract to acquire health knowledge to develop health beliefs and adopt health behaviors (18). Therefore, it is critical for community health education system to assess how they are meeting the public needs by discovering whether the public is satisfied with the products and services of health education provided by the community health centers. Carrying out an evaluation of satisfaction is conducive to finding the weak links in the development of health education, improving the quality and enhancing the attractiveness of community health education (19).

Previous studies have assessed the knowledge, utilization of and satisfaction with community health education and associated factors at individual level among residents in China (13, 14, 17, 20). The satisfactions in these studies are mainly evaluated through simple questions such as “do you think the current health education in your community can meet your needs”. However, little is known about the dimensions (perceived quality, perceived value, and public trust) of satisfaction with community health education and their correlation. Therefore, this study used customer satisfaction theory as a strategic tool to examine the relationship among the dimensions of satisfaction with community health education among residents in China.

## Methods

The design of this study is based on a classic customer model – American Customer Satisfaction Index (ACSI). This model is an econometric, causal model that links specific activities to perceptions of quality and satisfaction, which, in turn, are associated with specific behavioral responses, such as customer retention or complaints (21). There are five latent variables in our theoretical model, including public expectation (PE), perceived quality (PQ), perceived value (PV), public satisfaction (PS) and public trust (PT) (Table 1). Public expectation and perceived quality were used as antecedent variables in this study, because citizen satisfaction arises from a process in which citizens compare their perceptions of the performance of a community health education service against their prior expectations. And perceived quality refers to the overall evaluation of health education services in the community (21).

**Table 1:** Five dimensions of satisfaction with community health education and their definitions and key variables.

**Dimension**	**Definition**	**Variables**
Public expectation (PE)	An estimate of the quality of community health education service before it is available	Information content quality expectations The expectation of the quality of information communication Community service quality expectations
Perceived quality (PQ)	Evaluation and judgment of community health education service	Information content quality Quality of information communication Quality of community service
Perceived value (PV)	The perception of the benefits received after enjoying community health education service	Utilitarian value Hedonistic value
Public trust (PT)	Trust the community to be reliable and open to suggestions	Trust and support Degree of willingness to recommend
Public satisfaction (PS)	The actual experience is compared with the expected quality	Information quality satisfaction Information communication satisfaction Community service satisfaction

The presence of outcome variables of inherent interest is one of the key features of the ACSI model, including satisfaction and trust to community health education, which are seemed as targets for community health education services. Perceived value, as an intermediate variable, measures the subjective feelings of the public toward their own needs and the realization of their own interests after enjoying health education services provided by local communities. Public evaluation or attitude toward community health education services and their decision to finally take action depend on their perception of the value (22). The application of the ACSI model is intended to model how public expectation and perceived quality drive overall satisfaction and trust, in turn, how overall satisfaction and trust is related to outcomes of inherent interest to health educators, community administrators, and the public. Thus, overall satisfaction and trust with community health education remains the focus of the model, and the resulting estimates give information about both the drivers and consequences of satisfaction (or dissatisfaction) and trust (or distrust).

### Study Design and Sample

This is a cross-sectional study. An online survey was conducted among Chinese residents from April to June 2021. To achieve a heterogeneous sample, we required every investigator to use a quota sampling method to recruit a sample representative of the Chinese residents. The key demographic variables for quota sampling included gender, age and household registration type. That is, the number of male respondents was about equal to that of female ones, and at least one in six respondents were over 60 years. Assuming that about 47% of the population was satisfied with community health education based on the existing literature, with an alpha risk of 0.05, 451 individuals would provide a precision of 5% from the true values at 95% confidence level. The inclusion criteria include (1) being 12 years or older, (2) living in the local community for more than 60 days in the past year, (3) having adequate cognitive capacities to complete the survey, (4) willing to provide informed consent and to participate in the study. We excluded people who did not receive health education services. We recruited investigators to distribute the questionnaires until the demographic and sociological characteristics of the current sample were reasonable and representative.

### Data Collection

We recruited 10 volunteered investigators and each investigator was required to distribute and recover at least 30 questionnaires. Before the survey, investigators received standardized training. They conducted the investigation by sending the online questionnaire link to the residents who met the inclusion criteria. Each participant completed a self-administered online questionnaire independently, with investigator available to address the questions. A total of 581 questionnaires were sent out, and finally 474 valid questionnaires were collected. All participants were voluntary and their informed consents for participation in the survey were obtained prior to the questionnaire administration.

### Measures and Variables

We assessed the satisfaction with community health education by using a multifaceted instrument which was developed based on the existing literature and ACSI model. Final measures consisted of five parts including perceived quality, public expectation, perceived value, public trust and public satisfaction. We asked the participants to rate health education services in their communities according to their own feelings. Their responses were measured on a 5-point Likert scale from 1 (very disagree) to 5 (very agree). Public expectation contained 3 measurement variables, and the Cronbach's alphas was 0.884. Perceived quality consisted of four sub-dimensions (information quality, community service quality, health educators' service quality, and information spreading quality) with 34 measurement variables, and the Cronbach's alphas was 0.971. Perceived value contained 4 measurement variables, and the Cronbach's alphas was 0.889. Public satisfaction contained 3 measurement variables, and the Cronbach's alphas was 0.914. Public trust contained 4 measurement variables, and the Cronbach's alphas was 0.857.

We collected the basic characteristic variables of participants, including age (<20, 20–39, 40–59, ≥60 year), gender (male, female), marital status (married, single, separated/divorced/widowed), education (junior high school and below, high school or technical secondary school, junior college, bachelor, master and above), household registration type (non-agricultural, agriculture), and monthly household income per capita in Chinese Yuan (≤3,000, 3,000–6,000, 6,000–9,000, ≥9,000 CNY).

### Statistical Method

First, we obtained frequency (N) and percentage (%) statistics to show the basic characteristics of the participants. Second, we obtained mean (M) and standard deviation (SD) statistics to show the scores of satisfaction with community health education, and conducted one-way variance analysis to examine the differences in satisfaction scores of each dimension among residents with different characteristics. Third, we calculated the Pearson correlation coefficients to determine the associations among public expectations, perceived quality, perceived value, public satisfaction and public trust. Lastly, we employed a structural equation model (SEM) to verify the path and synthetic relationship among public expectation, perceived quality, perceived value, public satisfaction and public trust. Maximum likelihood estimation was performed to estimate these parameters in SEM. All statistical analyses were performed using SPSS 20 and AMOS 7. Statistical significance was set at *P* < 0.05.

## Results

### Basic Characteristics of Participants

Out of 474 participants, 52.9% were female, and 60.3% were non-agricultural household registration. The majority of them were 20–40 years old (52.2%) or 40–60 years old (29.2%). The percentage of respondents who were married and single was 48.0% and 48.6%, respectively. More than half of the participants had a bachelor's degree (54.8%). The percentage of these with a household monthly income per capita of ≤3,000 CNY per month was 21.8%, while that of ≥9,000 CNY was 22.6% (Table 2).

**Table 2:** Basic characteristics of participants in this study.

**Characteristics**		**Number (N)**	**Percentage (%)**
Gender			
	Male	223	47.1
	Female	250	52.9
Age in year			
	<20	45	9.5
	20–40	247	52.2
	40–60	138	29.2
	≥60	43	9.1
Household registration			
	Non-agricultural	285	60.3
	Agricultural	188	39.7
Marital status			
	Married	227	48.0
	Single	230	48.6
	Widowed or divorced	16	3.4
Education level			
	Junior high school and below	57	12.1
	High school or technical school	62	13.1
	Junior college	67	14.2
	Undergraduate	259	54.8
	Postgraduate	28	5.9
Household monthly income per capita in CNY			
	≤3,000	103	21.8
	3,000–6,000	178	37.6
	6,000–9,000	85	18.0
	≥9,000	107	22.6

*CNY, Chinese Yuan*.

### The Scores of Five Dimensions of Satisfaction With Community Health Education by Characteristics

The mean scores of public expectation, perceived quality, perceived value, public satisfaction, and public trust for the participants were 11.44 (total 15), 123.89 (total 170), 14.18 (total 20), 10.19 (total 15), and 15.61 (total 20), respectively. There were significant differences in the score of public expectation by age, marital status and household registration (*P* < 0.05). There were significant differences in the score of perceived quality by age, education level and household income (*P* < 0.05). There were significant differences in the score of perceived value by education level and household income (*P* < 0.05). There were significant differences in the score of public satisfaction by household registration (*P* < 0.05). There were significant differences in the score of public trust by age, education level and household income (*P* < 0.05) (Table 3).

**Table 3:** The scores of five dimensions of satisfaction with health education by characteristics (M, SD).

	**PE**	**PQ**	**PV**	**PS**	**PT**
Total	11.44 (2.77)	123.89 (20.13)	14.18 (2.97)	10.19 (2.99)	15.61 (2.66)
Gender
Male	11.51 (2.84)	125.29 (20.96)	14.30 (3.16)	10.44 (2.92)	15.60 (2.65)
Female	11.38 (2.71)	122.63 (19.32)	14.08 (2.80)	9.97 (3.05)	15.62 (2.67)
F value	0.26	2.06	0.62	2.99	0.01
P value	0.607	0.152	0.431	0.084	0.923
Age in year
<20	10.18 (3.19)	127.02 (17.99)	14.02 (3.04)	9.40 (2.97)	15.82 (2.53)
20–40	11.25 (2.81)	125.98 (21.01)	14.53 (2.97)	10.18 (3.01)	16.00 (2.58)
40–60	12.07 (2.51)	119.10 (19.53)	13.71 (3.019)	10.35 (3.07)	14.89 (2.73)
≥60	11.86 (2.30)	123.91 (16.58)	13.88 (2.61)	10.58 (2.60)	15.47 (2.61)
F value	6.39	3.93	2.47	1.42	5.38
P value	0.000	0.009	0.061	0.236	0.001
Marital status
Married	11.92 (2.61)	122.42 (21.96)	14.14 (3.15)	10.38 (3.16)	15.45 (2.92)
Single	10.95 (2.86)	125.73 (18.15)	14.30 (2.76)	10.03 (2.82)	15.83 (2.33)
Widowed/divorced	11.75 (2.57)	118.19 (18.45)	13.06 (3.36)	9.75 (3.00)	14.63 (2.96)
F value	7.21	2.21	1.34	0.96	2.29
P value	0.001	0.111	0.263	0.386	0.102
Education level
Junior high school and below	11.96 (2.51)	117.88 (18.32)	13.07 (3.14)	10.39 (2.81)	14.58 (2.79)
High school or technical school	10.89 (3.26)	121.66 (20.66)	13.74 (3.27)	9.71 (2.99)	15.06 (2.78)
Junior college	11.18 (2.94)	124.03 (20.63)	14.91 (2.77)	10.12 (3.14)	15.79 (2.61)
Undergraduate	11.46 (2.63)	126.15 (19.62)	14.40 (2.78)	10.36 (2.90)	16.00 (2.46)
Postgraduate	12.07 (2.80)	119.71 (23.52)	13.68 (3.54)	9.46 (3.78)	14.89 (3.25)
F value	1.66	2.62	3.98	1.10	4.91
P value	0.159	0.035	0.003	0.357	0.001
Household registration
Non-agricultural	10.74 (3.03)	121.85 (18.29)	14.16 (2.65)	9.83 (2.84)	15.39 (2.47)
Agricultural	11.91 (2.48)	125.23 (21.19)	14.20 (3.17)	10.43 (3.07)	15.75 (2.77)
F value	20.93	3.20	0.02	4.61	2.05
P value	<0.001	0.074	0.895	0.032	0.153
Household monthly income per capita
≤3,000	11.06 (2.91)	120.40 (16.90)	13.57 (2.56)	9.89 (2.78)	15.11 (2.38)
3,000–6,000	11.78 (2.70)	122.36 (20.14)	14.07 (2.95)	10.27 (2.94)	15.54 (2.71)
6,000–9,000	11.11 (2.83)	124.65 (19.33)	14.15 (2.86)	10.14 (3.08)	15.64 (2.63)
≥9,000	11.51 (2.66)	129.18 (22.64)	14.98 (3.33)	10.39 (3.22)	16.18 (2.77)
F value	2.00	3.95	4.19	0.55	2.93
P value	0.113	0.008	0.006	0.649	0.033

*PE, public expectation; PQ, perceived quality; PV, perceived value; PS, public satisfaction; PT, public trust. M, mean; SD, standard deviation. F value, one-way variance analysis*.

### Pearson Correlations Among Five Dimensions of Satisfaction With Community Health Education

Public expectations, perceived quality, perceived value, public satisfaction and public trust were significantly positively correlated with each other (*P* < 0.01). Out of the five dimensions of satisfaction of community health education, perceived quality had the strongest association with perceived value (r = 0.823), and public expectation had the weakest association with public trust (r = 0.165) (Table 4).

**Table 4:** Correlation among five dimensions of satisfaction with community health education.

	**PV**	**PT**	**PQ**	**PE**	**PS**
PV	1	0.643^**^	0.795^**^	0.168^**^	0.556^**^
PT	0.643^**^	1	0.700^**^	0.160^**^	0.483^**^
PQ	0.795^**^	0.700^**^	1	0.231^**^	0.645^**^
PE	0.168^**^	0.160^**^	0.231^**^	1	0.453^**^
PS	0.556^**^	0.483^**^	0.645^**^	0.453^**^	1

*PE, public expectation; PQ, perceived quality; PV, perceived value; PS, public satisfaction; PT, public trust*.

** *,P < 0.01*.

### Path Relationships Among Five Dimensions of Satisfaction With Community Health Education

**Figure 1:**
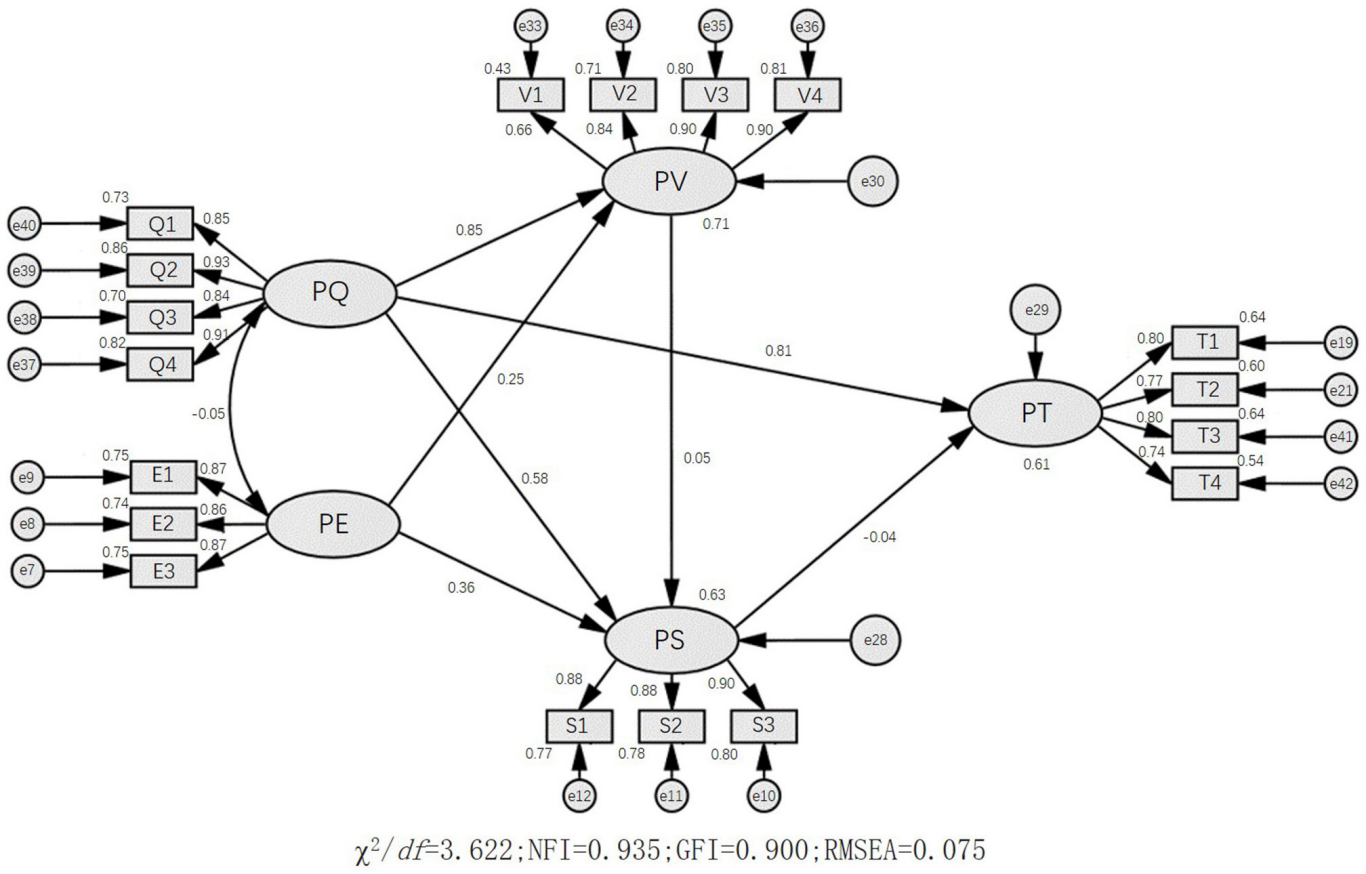
The path relationships among five dimensions of satisfaction with health education. PQ, perceived quality; PE, public expectation; PV, perceived value; PS, public satisfaction; PT, public trust.

The model fit indices of the SEM were all within specifications (GFI = 0.900, TLI = 0.942, IFI = 0.952, CFI = 0.921, NFI = 0.935, RMSEA = 0.075), and the chi-square to freedom degree ratio is 3.622, indicating good model fit. The edges between the dimensions in Figure 1 represent direct relationships. The indirect effect of one dimension on another is equal to the product of the regression coefficients of the two directly connected dimensions. For perceived quality, the standardized factor loadings ranged from 0.84 to 0.93. For public expectation, the standardized factor loadings ranged from 0.86 to 0.87. For perceived value, the standardized factor loadings ranged from 0.66 to 0.90. For public trust, the standardized factor loadings ranged from 0.74 to 0.80. For public satisfaction, the standardized factor loadings ranged from 0.88 to 0.90. There was a direct effect of perceived quality on perceived value (γ = 0.85, *P* < 0.01), public trust (γ = 0.81, *P* < 0.01) and public satisfaction (γ = 0.58, *P* < 0.01), and a direct effect of public expectation on public satisfaction (γ = 0.36, *P* < 0.01) and perceived value (γ = 0.25, *P* < 0.01).

## Discussion

This study presents critical information on the five dimension and their path relationships of satisfaction with community health education among residents in China. As a whole, the residents had low scores of satisfaction with community health education. Compared to their total scores, the mean scores of public expectation, perceived quality, perceived value, public satisfaction and public trust for the participants were rather low. The score of public satisfaction was the lowest when compared to the other four dimensions. There were significant differences in scores of five dimensions among the residents with different characteristics. By establishing a theoretical SEM on the basis of previous studies and achieving good model fit, we found that perceived quality had significant positive direct effects on perceived value, public trust and public satisfaction. Moreover, there was a significant indirect effect of public expectation on public satisfaction.

The findings showed that the public had low scores of satisfaction with community health education. This result is in line with other studies conducted in China. One study in Hebei province found that more than 50% of public were satisfied with community health education service (23). Among the five dimensions, the score of public trust was 15.61 out of total score 20. Previous studies had reported that 35.54%-40.91% of the public had high trust in health information from government health agencies (24). These findings suggest that there are still quite a few people who are not satisfied with community health education. It is probably because some individuals perceive that the content, format and approach of community health education service provided by government regulatory authority were not suitable or friendly. We also found that the level of public expectation was higher than that of public satisfaction. Therefore, policymakers should explore initiatives to strike a balance between public expectation and public satisfaction.

We found that many demographic features were associated with public expectation, perceived quality, perceived value, public satisfaction or public trust according to the results from the bivariate analyses. Rural residents had a higher level of public satisfaction than urban ones. It could be because the rural residents were more likely to use community health education, but urban residents tended to be picky about health education because they had more health knowledge (13). We also found that the residents of 40–60 year had higher public expectation, worse perceived quality, and lower public trust. Older residents are more dependent on community health education to gain health information than the younger. It may be because the older have greater need for health knowledge, but less confident in seeking health information. However, community health education fails to meet the needs of the older (23). Moreover, older people tend to live longer in the community and receive more public health services, such as chronic disease management. All the above remind us that community health education should focus on the needs of the older. A bottom-up approach to assess community needs of health education can not only improve the satisfaction, but also led to the implementation of effective interventions using the resources of the community (25).

This study found public satisfaction is directly affected by public expectation, which is consistent with Oliver and Susarla's theory (26, 27). Expectation is a set of beliefs that a customer possesses about the products or services (27), and the difference between pre-consumption belief and post-consumption experience is known as a confirmation of satisfaction. The public expect benefits from the community health education service (28, 29). When there is a gap between their expectations and the actual health education services they receive, the public are dissatisfied with the services. We also found perceived quality had a significant positive direct effect on public satisfaction, perceived value and public trust. This is also in line with the Information System Success Model and ACSI (26, 30, 31). Therefore, perceived quality is an important dimension, and it is critical to improving the quality of community health education. Effective measures, such as providing more friendly services, and improving the form and content of health education, should be taken to improve the perceived quality. Higher quality of health education needs higher quality of primary health educators (32). It is necessary to build core competencies of community health educators to fill their roles in community health education (13).

Our study found that there are still some problems in community health education program. Firstly, equalization of China's Basic Public Health Services (BPHS) emphasizes providing BPHS in response to residents' needs rather than providing the same BPHS to everybody (11). However, expectation and perceived quality for community health education vary from person to person. Therefore, effective health education should include a health needs assessment first and then design the content in accordance with residents' health knowledge needs (11). We are now advocating the strengthening of people-centered health communication strategies (2). If there are no comprehensive community health needs assessment, the content and approach of health education could not be responsive to residents' health needs. Secondly, improving the quality of community health educators and providing financial support is the key to improving the quality of health education. Most community health educators are part-time workers, and consisted of many community nurses and only a few public health specialists (16, 33–35). If we want to attract and retain qualified health educators in community health institutions, we may need to improve working conditions, income, and social security.

Public satisfaction measurement model of community health education in this study has several practical implications. First, it offers ideas for community health educators on how to improve effective health education so that the public are more prone to follow their advices and are more responsible for their own health (36). We call on the community to pay attention to the perceived quality of citizen which is critical to the public trust and satisfaction. Second, we provide a direction for how to develop the core competence of community health educators, which should include information content quality, quality of information communication and quality of service. Third, satisfaction survey can assess the quality of local government services. Therefore, we provided a assess tool to help health sectors to find weakness and deficiencies in health education so that they could improve targeted services. To date, the level of public satisfaction with community health education was low due to many reasons, but it would be improved if we will take targeted measures.

There are several limitations to acknowledge in this study. First, the data were obtained by using self-reported questionnaires, which may result in recalling bias of information. Second, although ACSI model is widely used in government service satisfaction, it has not been applied in the field of health education. Third, this is a cross-sectional study, so the causal relationship among five dimensions of satisfaction can't be identified. Third, our questionnaire survey was conducted by using online surveys, which might have created a selection bias. Some people who were not good at using electronic products were easily overlooked, especially the old. To minimize the bias, we made sure that the number of male respondents was about equal to that of female ones, and at least one in six respondents were over 60 years.

In conclusion, we provide a good tool to measure satisfaction with community health education, and find many residents had low scores of satisfaction with health education provided by community health centers in China. We obtained a structural equation model with good fitting degree. Perceived quality had significant positive direct effects on perceived value, public trust and public satisfaction. Public expectation and perceived value made positive contributions to public satisfaction. The tool we developed in this study can help assess the public satisfaction level and identify the management strategies to improve public satisfaction, match public desires and promote the use of community health education.

## Data Availability Statement

The raw data supporting the conclusions of this article will be made available by the authors, without undue reservation.

## Ethics Statement

The studies involving human participants were reviewed and approved by Research Office of Shaanxi Institute of International Business. Written informed consent to participate in this study was provided by the participants' legal guardian/next of kin. Written informed consent was obtained from the individual(s), and minor(s)' legal guardian/next of kin, for the publication of any potentially identifiable images or data included in this article.

## Author Contributions

YT, HW, and YW conceived and designed the study. YT, KZ, HZ, YQ, JG, and YM collected the data. YT and QL did the data analysis. YT wrote the first draft. HW revised and edited the manuscript. XS and YW supervised the manuscript. All authors approved the final manuscript submitted for publication.

## Funding

This work was supported by Cheeloo College of Medicine, Shandong University (QLYXJY-201946), the National Key Research and Development Program of China (2020YFC2006505), and the Scientific Research Project of Shaanxi Provincial Education Department in 2021- the key research base project of philosophy and social sciences (21JZ017).

## Conflict of Interest

The authors declare that the research was conducted in the absence of any commercial or financial relationships that could be construed as a potential conflict of interest.

## Publisher's Note

All claims expressed in this article are solely those of the authors and do not necessarily represent those of their affiliated organizations, or those of the publisher, the editors and the reviewers. Any product that may be evaluated in this article, or claim that may be made by its manufacturer, is not guaranteed or endorsed by the publisher.
